# Macrophage Membrane‐Camouflaged Nanozyme Microneedles Restore Immunovascular Homeostasis Through SPP1‐ApoE Signaling in Diabetic Wounds

**DOI:** 10.1002/adhm.71456

**Published:** 2026-07-26

**Authors:** Qipeng Wu, Yuan Xiong, Li Lu, Han Wang, Hui Hu, Mohammad‐Ali Shahbazi, Guohui Liu, Bobin Mi

**Affiliations:** ^1^ Department of Orthopedics Union Hospital Tongji Medical College Huazhong University of Science and Technology Wuhan China; ^2^ Department of Orthopedics Tongji Hospital Huazhong University of Science and Technology Wuhan Hubei China; ^3^ Department of Rehabilitation Tongji Hospital Tongji Medical College Huazhong University of Science and Technology Wuhan China; ^4^ Department of Anesthesiology, Department of Anesthesiology, Peking University People's Hospital, Women and Children's Hospital Qingdao University Qingdao China; ^5^ Department of Biomaterials and Biomedical Technology Personalized Medicine Research Institute (PRECISION) University Medical Center Groningen (UMCG) University of Groningen Groningen The Netherlands; ^6^ Longly Biotechnology (Wuhan) Co., Ltd Wuhan China

**Keywords:** angiogenesis, macrophage, metal‐organic frameworks, nanozyme, wound healing

## Abstract

Chronic diabetic wounds are characterized by persistent inflammation, impaired angiogenesis, and disrupted intercellular communication, including altered macrophage‐endothelial interactions. In this study, we develop macrophage membrane‐camouflaged, didymin (DM)‐loaded metal‐organic framework microneedles (Mac@DM‐MOF MNs), a biomimetic nanozyme platform designed to selectively restore this intercellular communication and re‐establish a pro‐regenerative microenvironment. RNA‐seq and mechanistic analyses identify activation of the SPP1‐ApoE signaling axis, an unrecognized pathway linking M2 polarization to endothelial activation, as a central mechanism by which Mac@DM‐MOF MNs synchronize inflammation resolution and angiogenesis. Nanozyme‐mediated ROS scavenging relieves redox stress, while DM promotes macrophage polarizationtoward the reparative M2 state. M2‐derived SPP1 subsequently interacts with endothelial ApoE, thereby promoting endothelial sprouting, and restoring vascular functionality. In diabetic mouse and Bama mini pig models, Mac@DM‐MOF MNs attenuate inflammation, rescue angiogenic deficits, and markedly accelerate wound closure. Importantly, vascular restoration further reinforces M2 polarization, forming a self‐sustaining pro‐healing feedback loop. Our findings define macrophage‐endothelial coupling as a pivotal regulatory mechanism in diabetic wound repair and introduce a synergistic nanomedicine‐based strategy that concurrently resolves chronic inflammation and restores angiogenesis.

## Graphical abstract

Macrophage membrane‐coated Cu/Zn‐MOF nanozyme microneedles (MNs) reprogram the diabetic wound microenvironment through localized, minimally invasive delivery. By suppressing M1‐like macrophage activation while promoting extracellular matrix deposition and angiogenesis, this biomimetic platform accelerates diabetic wound repair. The strategy integrates immune modulation, redox‐active MOF therapeutics, and microneedle‐based delivery to enhance tissue regeneration.

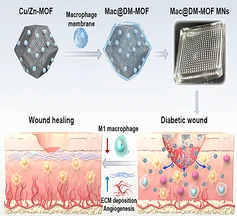

## 1 Introduction

Diabetic foot ulcers (DFUs) represent one of the most debilitating complications of diabetes mellitus, characterized by a microenvironment dominated by chronic inflammation, excessive oxidative stress, and profoundly impaired angiogenesis [1, 2]. These pathological features collectively establish a nonresolving inflammatory state that resists standard interventions‐including debridement, grafting, and negative‐pressure therapieswhich largely provide symptomatic relief without addressing the mechanistic origins of healing failure [3, 4]. The inability to restore coordinated immune and vascular responses results in persistent ulceration, recurrent infections, and high amputation risk [5, 6]. Effective diabetic wound repair therefore requires reconstruction of the immunovascular regulatory circuit that orchestrates inflammation resolution and tissue regeneration.

Macrophages are principal sensors and regulators of the wound niche, transitioning from a pro‐inflammatory M1 phenotype to a reparative M2 phenotype in response to local cues [7, 8, 9]. This transition is essential for suppressing inflammatory cytokines, stimulating extracellular matrix deposition, and, critically, initiating angiogenesis. However, in diabetic wounds, sustained hyperglycemia and ROS accumulation disrupt macrophage metabolic homeostasis, delaying or preventing M2 polarization [10].

Macrophages and endothelial cells(ECs) engage in reciprocal crosstalk that contributes to the coordination of inflammatory resolution and angiogenic responses during wound repair [11, 12]. Diabetic ECs exhibit blunted VEGFR2 signaling, mitochondrial dysfunction, and impaired sprouting capacity, contributing to vascular rarefaction and tissue hypoxia [13, 14]. Together, these defects indicate that macrophage‐endothelial crosstalk is a central point of failure in diabetic wound healing, yet the molecular pathways coupling macrophage phenotype to EC activation remain insufficiently defined.

Although numerous biomaterial and drug‐based therapies aim to modulate macrophage phenotype or stimulate angiogenesis, several challenges persist [15, 16]. First, low bioavailability and degradation under oxidative conditions limit efficacy in the diabetic wound niche. Second, restricted targeting capacity prevents sustained engagement with resident macrophages and ECs. In addition, lack of mechanisms for continuous, low‐dose modulation, critical for reprogramming chronically inflamed tissues [17, 18]. These limitations highlight the need for a delivery system capable of precise targeting, oxidative stress regulation, and coordinated modulation of immunovascular interactions [19, 20].

Metal‐organic frameworks (MOFs) have emerged as versatile nanoplatforms for biomedical applications due to their high porosity, tunable catalytic activity, and responsiveness to microenvironmental cues [21, 22]. ROS‐responsive MOFs can function as nanozymes that degrade oxidative intermediates, thereby restoring redox balance‐an essential prerequisite for macrophage metabolic reprogramming [4, 23]. In diabetic wounds, localized ROS modulation has been shown to promote M2 polarization and enhance angiogenesis [24, 25, 26]. To overcome the challenges of targeted delivery, macrophage membrane coating has been explored as a biomimetic surface‐engineering strategy to enhance nanoparticle compatibility with the inflammatory microenvironment and facilitate interactions with relevant immune cells. Integrated into a microneedle (MN) system, this biomimetic nanoplatform enables minimally invasive, localized, and sustained delivery directly into the wound bed [27].

**SCHEME 1:**
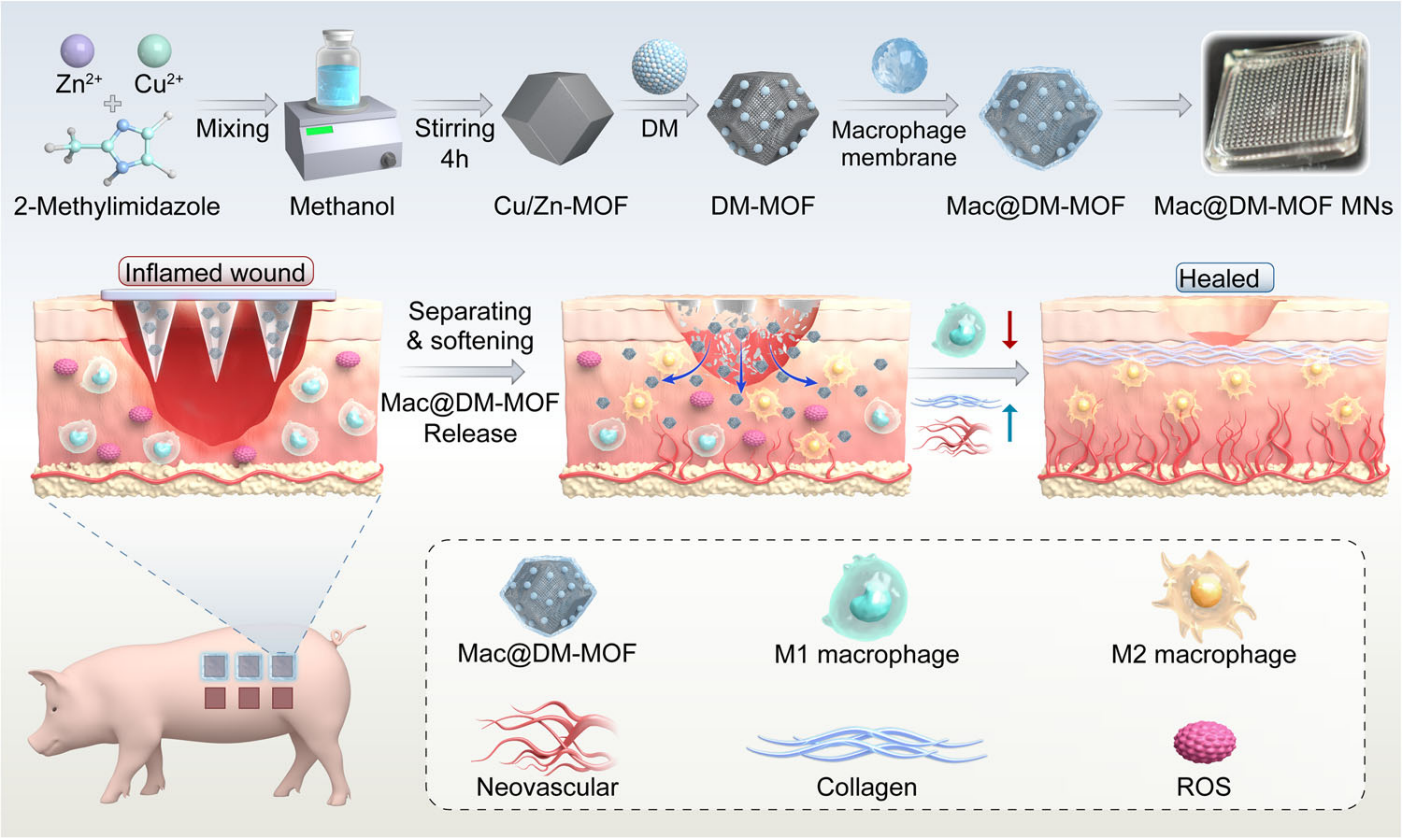
Schematic illustration for the construction of Mac@DM‐MOF MNs and their mechanism for promoting diabetic wound healing. Upon application to the wound site, the MN patch releases Mac@DM‐MOF, precisely targeting macrophages within the wound. This system efficiently scavenges intracellular ROS and induces macrophage polarization toward an anti‐inflammatory phenotype, thereby alleviating the local inflammatory microenvironment. The improved inflammatory state subsequently facilitates angiogenesis, which, in turn, restores local blood supply to the wound. This restoration further enhances the anti‐inflammatory polarization of macrophages, establishing a positive feedback loop within the wound microenvironment. This synergistic interaction ultimately accelerates wound healing with high efficiency.

Didymin (DM), a flavonoid capable of promoting M2 polarization through metabolic reprogramming, further augments the therapeutic potential of MOFs [28, 29]. Building on these principles, we engineered macrophage membrane‐coated, didymin‐loaded MOF microneedles (Mac@DM‐MOF MNs)‐a multifunctional therapeutic platform designed to (i) recalibrate macrophage polarization under oxidative stress, (ii) rescue endothelial angiogenic dysfunction, and (iii) reconstruct macrophage–endothelial crosstalk at diabetic wound sites (Scheme 1). Using diabetic mouse and Bama mini pig models, we demonstrate that Mac@DM‐MOF MNs effectively resolve oxidative stress, attenuate inflammation, restore angiogenic capacity, and significantly accelerate wound closure.

## 2 Results and Discussion

### 2.1 Physicochemical Characterization of Mac@DM‐MOF

**FIGURE 1:**
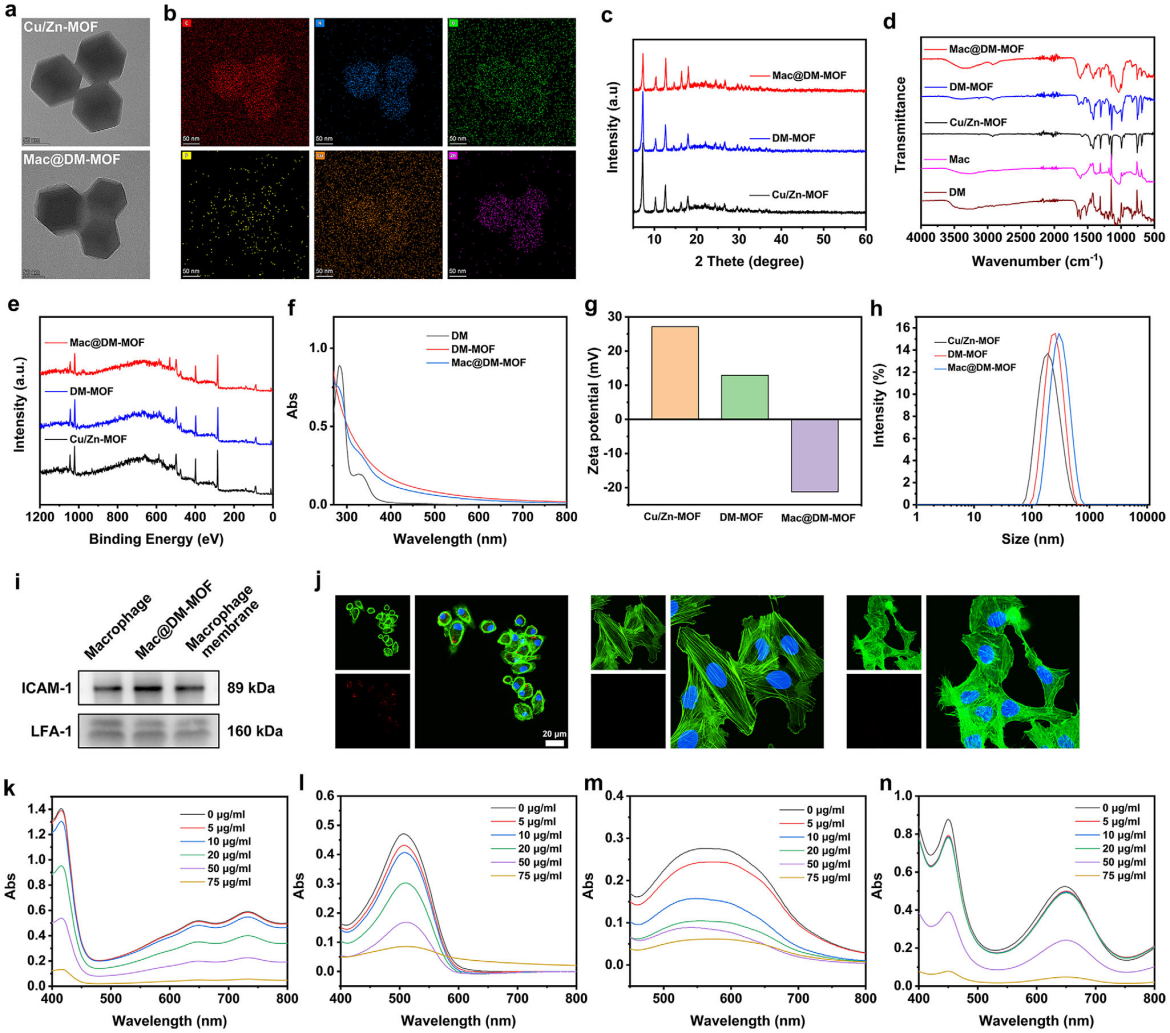
Structural and functional properties of Mac@DM‐MOF. (a). TEM imaging showed the uniform dispersion of Cu/Zn‐MOF in a rhombic dodecahedral structure and membrane‐coating morphology of Mac@DM‐MOF nanoparticles. (b). Elemental mapping confirming the homogeneous distribution of C, N, O, P, Zn, and Cu within the Mac@DM‐MOF. (c). Powder XRD patterns of Cu/Zn‐MOF, DM‐MOF, and Mac@DM‐MOF, demonstrating preserved crystalline structure. (d). FT‐IR spectra of Cu/Zn‐MOF, DM‐MOF, and Mac@DM‐MOF, indicating successful drug encapsulation and membrane surface coverage. (e). XPS analysis validating the synthesis of the composite nanoparticles. f. UV‐Vis absorption spectra of DM, DM‐MOF, and Mac@DM‐MOF. (g). Zeta potential of MOF, DM‐MOF, and Mac@DM‐MOF. (h). Size distribution analysis for Cu/Zn‐MOF, DM‐MOF, and Mac@DM‐MOF, revealing uniform particle sizes. (i). Detection of surface markers on different particles. (j). Phagocytosis efficiency of Mac@DM‐MOF in various cell types. (k). ABTS•^+^ radical scavenging assay. (l). Nitric oxide radical (•NO) scavenging assay. (m). Superoxide anion (•O_2_^−^) scavenging assay. (n). Hydroxyl radical (•OH) scavenging assay.

Transmission electron microscopy (TEM) images revealed that the synthesized Cu/Zn‐MOF nanoparticles exhibited a uniform rhombic dodecahedral structure and the Mac@DM‐MOF showed a membrane‐coating morphology (Figure 1a). Elemental mapping further confirmed the homogeneous distribution of C, N, O, P, Zn, and Cu within the Mac@DM‐MOF framework (Figure 1b). Powder x‐ray diffraction (XRD) patterns demonstrated that DM‐MOF and Mac@DM‐MOF retained the characteristic diffraction peaks of Cu/Zn‐MOF, indicating that neither the drug encapsulation nor the membrane coating compromised the crystalline structure of the MOF (Figure 1c). Key vibrational peaks of Cu/Zn‐MOF were observed in the FT‐IR spectra, including the C═N stretching vibration peak at 1580 cm^−^^1^, Zn─O coordination bonding at 755 cm^−^^1^, and Zn‐N coordination bonding at 691 cm^−^^1^. Moreover, DM‐MOF exhibited an attenuated O─H bonding absorption peak at 3400 cm^−^^1^, along with weakened C─O and C═O bonding absorption peaks at 1260 and 1640 cm^−^^1^, respectively. These changes indicate the successful encapsulation of DM within the Cu/Zn‐MOF pores. FT‐IR spectra of Mac@DM‐MOF revealed an additional C═O absorption peak at 1606 cm^−^^1^, attributed to the successful surface encapsulation by macrophage membrane (Figure 1d). X‐ray photoelectron spectroscopy (XPS) provided further evidence of successful composite synthesis. Peaks corresponding to Zn 2p (1021.6 eV) and Cu 2p (933.9 eV) were detected in the spectra of Cu/Zn‐MOF, DM‐MOF, and Mac@DM‐MOF (Figure 1e), validating the preservation of the Cu/Zn‐MOF framework during the functionalization. The UV‐vis absorption spectrum of Mac@DM‐MOF showed a characteristic DM absorption peak at 283 nm, confirming effective drug loading (Figure 1f). Surface charge measurements revealed a zeta potential shift from +12.9 for DM‐MOF to ‐21.2 mV for Mac@DM‐MOF, attributed to the introduction of membrane composition (Figure 1g). The hydrodynamic sizes of DM‐MOF and Mac@DM‐MOF, measured via dynamic light scattering (DLS), were 223.5 nm and 276 nm respectively, consistent with the observed surface modifications (Figure 1h).

To investigate the targeting ability of macrophage membrane‐coated nanoparticles, western blot (WB) analysis confirmed the successful incorporation of macrophage membrane markers onto DM‐MOF (Figure 1i). Cellular uptake studies further demonstrated that Mac@DM‐MOF nanoparticles exhibited the enhanced macrophage targeting with minimal uptake by endothelial and fibroblast cells (Figure 1j). These results suggest a strong macrophage‐targeting capability of Mac@DM‐MOF nanoparticles.

The antioxidant capacity of Mac@DM‐MOF was evaluated against various reactive oxygen species (ROS) under aqueous conditions. Using radical cation (ABTS•^+^) of 2,2’‐azinobis (3‐ethylbenzothiazoline‐6‐sulfonic acid) as a standard free radical model, scavenging activities were assessed at varying concentrations (5, 10, 25, 50, and 75 µg/mL) in PBS (pH 5.5) over 30 min, simulating an acidic ischemic microenvironment. The Mac@DM‐MOF displayed a concentration‐dependent quenching effect on ABTS•^+^, evidenced by a progressive reduction in the absorption peak at 734 nm (Figure 1k). Further assessments were conducted to target physiologically relevant ROS, including nitric oxide radicals (•NO), superoxide anions (•O_2_^−^), and hydroxyl radicals (•OH). The •NO scavenging ability was evaluated using Griess reagents, revealing a concentration‐dependent decrease in nitrite/nitrate‐associated absorbance at 507 nm (Figure 1l). Superoxide dismutase (SOD)‐like activity assays demonstrated that Mac@DM‐MOF effectively scavenged •O_2_^−^ radicals via a xanthine oxidase reaction system (Figure 1m). Similarly, the •OH scavenging capacity was confirmed through Fenton reaction assays, where increasing concentrations of Mac@DM‐MOF resulted in a reduction of the pink absorbance associated with •OH, indicating effective antioxidant activity (Figure 1n). Collectively, these results highlight the structural stability, drug encapsulation efficiency, macrophage‐targeting ability, and robust antioxidant performance of Mac@DM‐MOF nanoparticles, making them suitable for biomedical applications.

### 2.2 Characterization of Mac@DM‐MOF MNs

**FIGURE 2:**
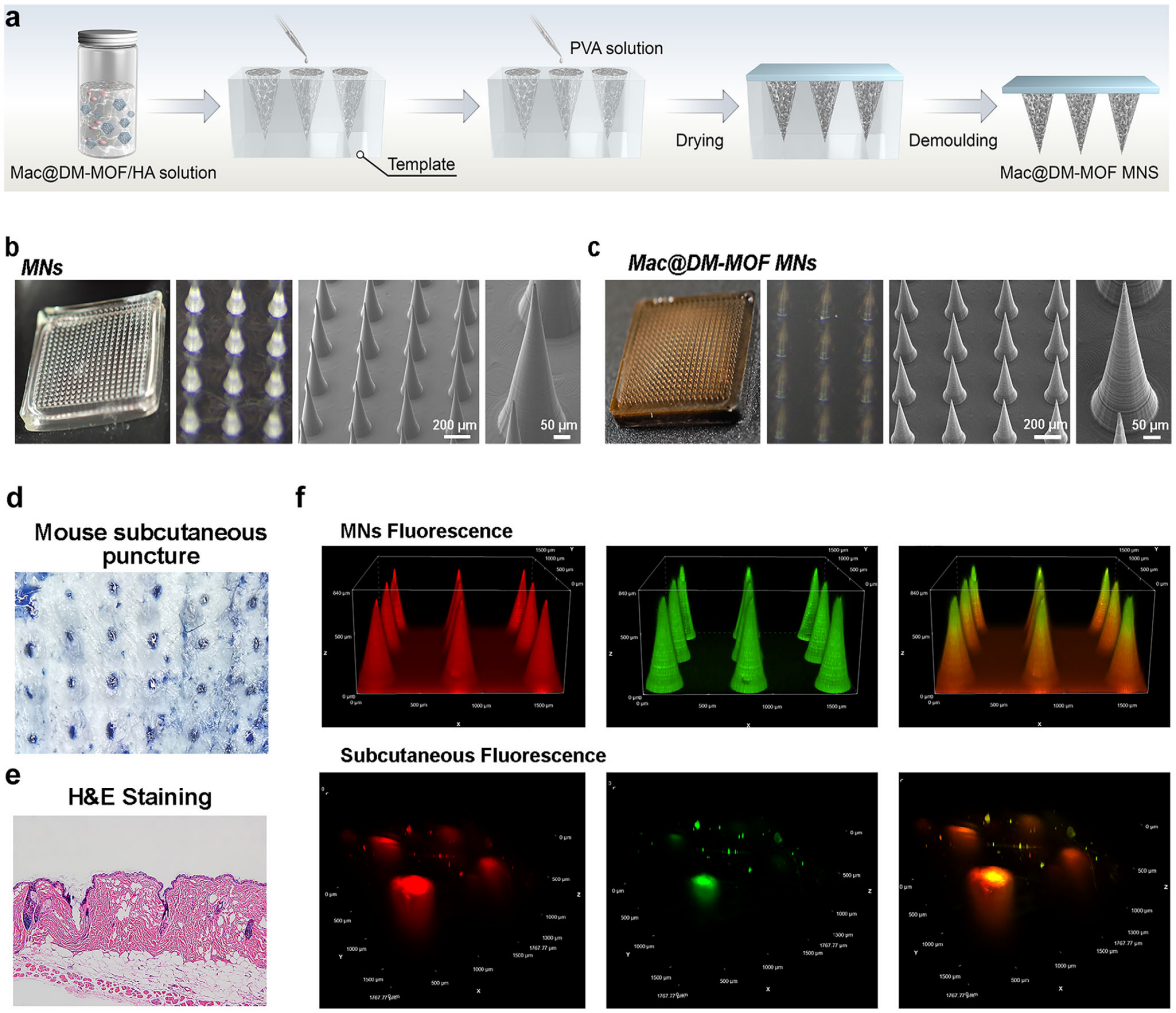
Integration of Mac@DM‐MOF into HA‐based MN patches for enhanced bioavailability.(a) Schematic illustration depicting the fabrication process of Mac@DM‐MOF MNs. (b, c) SEM and optical stereomicroscope images showing the structure of the MNs and Mac@DM‐MOF MNs. (d, e) Trypan blue (d) and H&E staining (e) images of mouse skin following the application of Mac@DM‐MOF MNs, confirming successful skin penetration. (f) Fluorescence imaging of Mac@DM‐MOF MNs, illustrating their ability to penetrate tissue and degrade within 1 h post‐application.

To enhance the bioavailability and localized delivery of Mac@DM‐MOF, we developed HA‐based MN patches incorporating Mac@DM‐MOF. The MNs were fabricated using a two‐layer molding strategy with a polydimethylsiloxane (PDMS) mold and were loaded with various nanomaterials, including MOF, DM‐MOF, and Mac@DM‐MOF (Figure 2a). Scanning electron microscopy (SEM) and optical stereomicroscope imaging confirmed that the MN patches featured cone‐shaped needles with sharp, well‐defined tips and uniform dimensions. High‐resolution SEM images revealed distinct morphological differences between the blank MNs and the Mac@DM‐MOF MNs. While the blank MNs displayed a smooth surface, the tips of the Mac@DM‐MOF MNs exhibited an irregular and rough texture, indicative of successful loading of the nanoparticles (Figure 2b,c).

The force‐displacement curve showed that Mac@DM‐MOF MNs exhibited high compressive strength as the displacement increased, which was sufficient for the penetration of skin, as seen by the displacement of 0.4 mm with approximately 0.4 N per needle (Figure S1). To further evaluate the mechanical strength and skin penetration capability of the Mac@DM‐MOF MNs, trypan blue staining (Figure 2d) and hematoxylin and eosin (H&E) staining (Figure 2e) were performed on mouse skin upon post‐application. These results demonstrated that the Mac@DM‐MOF MNs possessed sufficient strength to effectively penetrate the epidermal barrier without causing obvious tissue disruption.

Fluorescence imaging further validated the functionality of the MN patches. The MNs were labeled with fluorescein isothiocyanate (FITC), while Mac@DM‐MOF nanoparticles were labeled with rhodamine B (RhB). Following applications, fluorescence signals confirmed successful penetration of the MNs into the tissues and indicated progressive degradation of the patches within 1 h of treatment (Figure 2f). This rapid degradation ensures effective release and bioavailability of the loaded Mac@DM‐MOF at the targeted site, highlighting the potential of HA‐based MNs for efficient and controlled nanoparticle delivery. Then, the cumulative release profile of DM from macrophage membrane‐coated MOF nanoparticles was determined, indicating that the discharge rate of Mac@DM‐MOF immersed in PBS was comparable to that treated with H_2_O_2_. Notably, the nano‐system released 48.2% ± 3.89% and 68.68% ± 2.42% of DM at pH 7.4 and 5.5 respectively within 12 h. After 48 h, the release content of 77.6% ± 2.13% at pH 7.4 and 88.68% ± 2.56% at pH 5.5 was observed, demonstrating the pH‐responsive character of Mac@DM‐MOF, enabling the timely and sustainable entrance of DM into the acid diabetic wound microenvironment (Figure S2).

### 2.3 Mac@DM‐MOF Modulates Macrophage Polarization and Alleviates Inflammation

**FIGURE 3:**
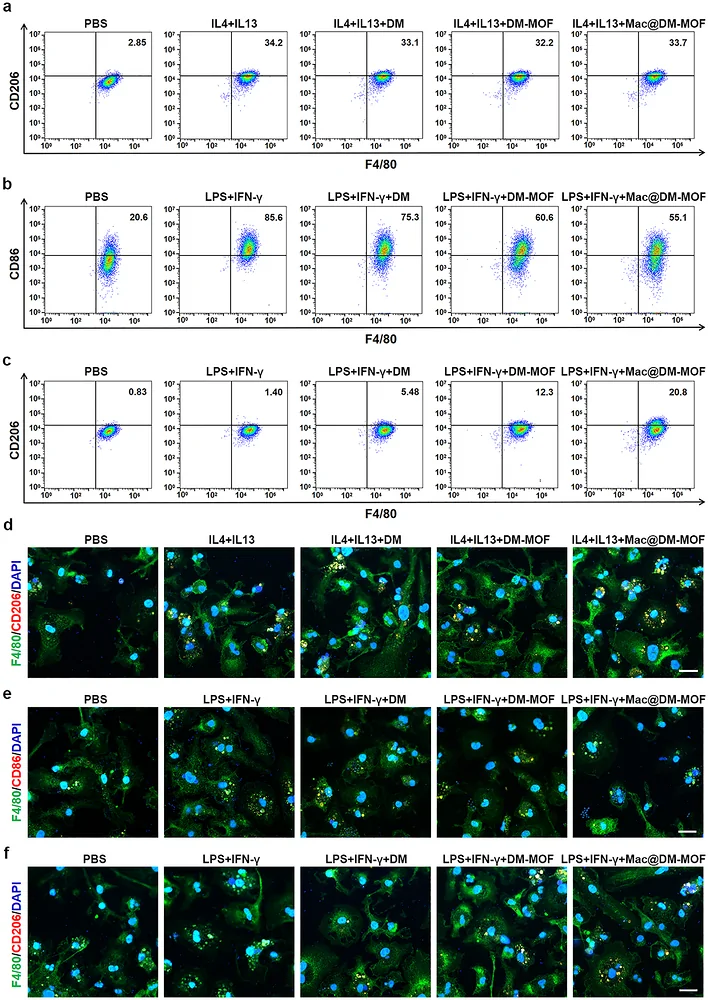
Effects of Mac@DM‐MOF on macrophage phenotype polarization. (a). M2 phenotype changes in macrophages pre‐treated with IL‐4 and IL‐13 as detected by flow cytometry across different treatment groups. (b). M1 phenotype changes in macrophages pre‐treated with LPS and IFN‐γ as detected by flow cytometry across different treatment groups. (c). M2 phenotype changes in macrophages pre‐treated with LPS and IFN‐γ as detected by flow cytometry across different treatment groups. (d). M2 phenotype changes in macrophages pre‐treated with IL‐4 and IL‐13 as detected by immunofluorescence across different treatment groups, scale bar: 20 µm. (e). M1 phenotype changes in macrophages pre‐treated with LPS and IFN‐γ as detected by immunofluorescence across different treatment groups, scale bar: 20 µm. (f). M2 phenotype changes in macrophages pre‐treated with LPS and IFN‐γ as detected by immunofluorescence across different treatment groups, scale bar: 20 µm.

Next, we investigated the biological effects of the synthetic nanozyme system at the cellular level. We first evaluated the cytotoxicity of various nanomaterials on human umbilical vein endothelial cells (HUVECs) and bone marrow‐derived macrophages (BMDMs) using the CCK‐8 assay and live/dead cell staining. As shown in Figures S3–S5, the Mac@DM‐MOF components exhibited minimal cytotoxicity toward both cell types, with no statistically significant differences compared to the control groups. This low toxicity provides a crucial foundation for their subsequent biological applications. We then investigated the in vitro effects of Mac@DM‐MOF on macrophage polarization and the secretion of inflammatory cytokines. Initially, macrophages were pre‐stimulated with IL‐4 and IL‐13, followed by treatment with PBS, DM, DM‐MOF, or Mac@DM‐MOF for 48 h. Flow cytometry analysis revealed that intervention of DM and MOF failed to affect the M2 anti‐inflammatory phenotype induced by IL‐4 and IL‐13 (Figures 3a and S6). To further examine the impact of Mac@DM‐MOF in an inflammatory microenvironment, macrophages were exposed to LPS and IFN‐γ to simulate inflammation in vitro. Following treatment with PBS, DM, DM‐MOF, or Mac@DM‐MOF for 48 h, flow cytometry analysis indicated that the DM partially reversed the M1 inflammatory phenotype, which was enhanced by delivery route improvement (Figures 3b and S7). Among the groups, Mac@DM‐MOF exhibited the most pronounced reversal, achieving a statistically significant difference compared to the DM‐MOF group (p = 0.0490). Additionally, the levels of M2 phenotype markers were assessedshowing a marked increase in M2 marker expression in the material‐treated groups, particularly in the Mac@DM‐MOF group, which was much higher than that of the DM‐MOF group (p = 0.0114) (Figures 3c and S8).

To validate these findings, immunofluorescence staining was performed. As shown in Figures 3d and S9, material‐treated cells displayed substantial polarization toward the M2 anti‐inflammatory phenotype (CD206), with a statistically significant difference compared to the control group. Similarly, in macrophages pre‐stimulated with LPS and IFN‐γ, the immunofluorescence results aligned well with those of flow cytometry studies. As depicted in Figures 3e and S10, the Mac@DM‐MOF group demonstrated the most significant reversal of the M1 inflammatory phenotype (p = 0.0011). Furthermore, M2 phenotype marker expression (CD206) was elevated in material‐treated cells, with the highest levels observed in the Mac@DM‐MOF group (p < 0.0001) (Figures 3f 3c and S11). These results collectively indicate that Mac@DM‐MOF effectively modulates macrophage polarization, improves the inflammatory microenvironment, and promotes a pro‐healing response.

### 2.4 Mac@DM‐MOF Mitigate Inflammation Via Degradation of ROS

**FIGURE 4:**
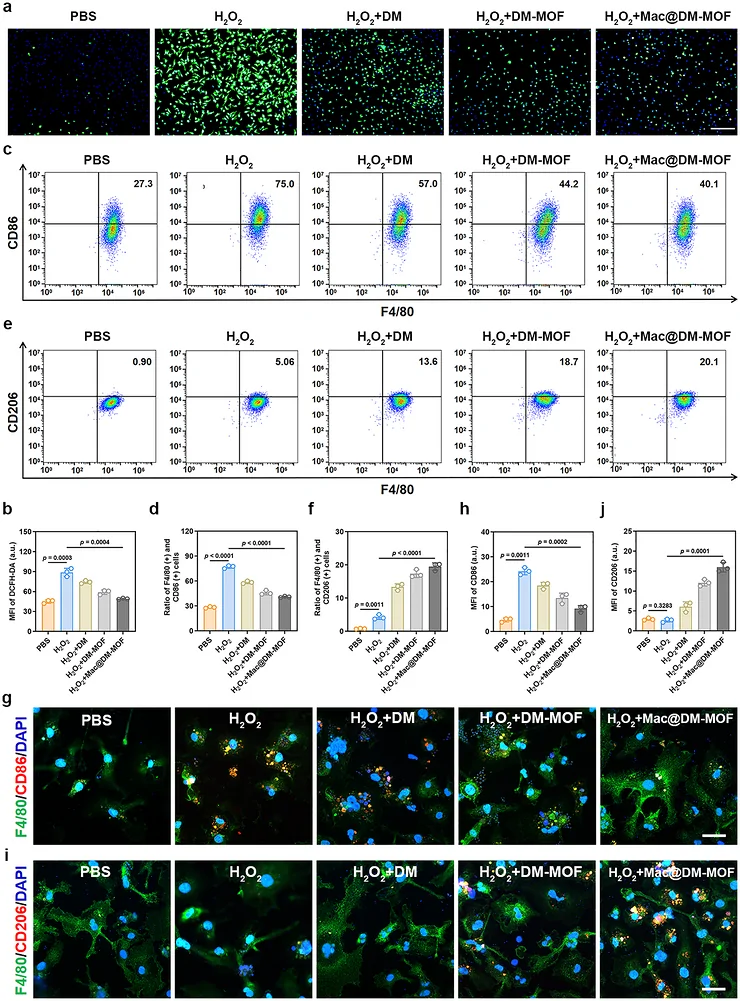
Mac@DM‐MOF mitigate inflammation by degrading ROS. (a, b). Immunofluorescence analysis showing the ROS degradation effects of various materials, along with statistical results, scale bar: 20 µm. (c, d). Expression levels of macrophage M1 phenotype across different treatment groups. (e, f). Expression levels of macrophage M2 phenotype across different treatment groups. (g, h). Immunofluorescence analysis showing the impact of various materials on macrophage M1 phenotype polarization under high oxidative stress, with corresponding statistical data, scale bar: 20 µm. (i, j). Immunofluorescence analysis showing the impact of various materials on macrophage M2 phenotype polarization under high oxidative stress, with corresponding statistical data, scale bar: 20 µm.

Excessive oxidative stress is a hallmark of diabetic wounds, contributing to their impaired healing. To investigate the impact of Mac@DM‐MOF on oxidative stress, we established an oxidative stress model in macrophages by treating them with H_2_O_2_, followed by the assessment of intracellular ROS levels using 2,7‐dichlorodihydrofluorescein diacetate (DCFH‐DA) fluorescent probes. As shown in Figure 4a,b, the nanozyme system mitigated H_2_O_2_‐induced oxidative stress, markedly reducing ROS levels in macrophages. Notably, the Mac@DM‐MOF group exhibited the most substantial ROS degradation (p = 0.0004). We further explored the effects of these systems on the inflammatory microenvironment. Flow cytometry analysis revealed that the nanozymes effectively reversed H_2_O_2_‐induced polarization of macrophages toward the inflammatory M1 phenotype, with Mac@DM‐MOF demonstrating the most pronounced effect (p < 0.0001, Figure 4c,d). Additionally, we measured M2 phenotype markers in macrophages treated with different materials. As shown in Figure 4e,f, the levels of M2 markers were elevated in cells treated with the nanozymes, with Mac@DM‐MOF inducing the highest levels (p < 0.0001). These findings suggest that the nanozymes reprogram macrophage polarization to reshape the oxidative stress microenvironment.

To corroborate these findings, we performed immunofluorescence staining. As shown in Figure 4g,h, the nanosystems notably ameliorated macrophage inflammatory polarization under oxidative stress, where Mac@DM‐MOF exhibited the highest improvement (p = 0.0002). Furthermore, the nanosystems effectively induced anti‐inflammatory macrophage polarization, with Mac@DM‐MOF showing the strongest effect (p = 0.0001, Figure 4i,j). In addition, we evaluated the secretion of inflammatory cytokines IL‐6 and IFN‐γ in macrophages pre‐treated with H_2_O_2_ and subjected to different material treatments. As shown in Figure S12, Mac@DM‐MOF reduced the secretion of both cytokines, with statistically significant differences (p = 0.0009 for IL‐6 and p < 0.0001 for IFN‐γ). These results firmly demonstrate that Mac@DM‐MOF exerts potent anti‐inflammatory effects by effectively degrading ROS, thereby improving the oxidative stress microenvironment.

### 2.5 Activation of SPP1/ApoE Signaling Pathway as a Potential Mechanism for Mac@DM‐MOF

**FIGURE 5:**
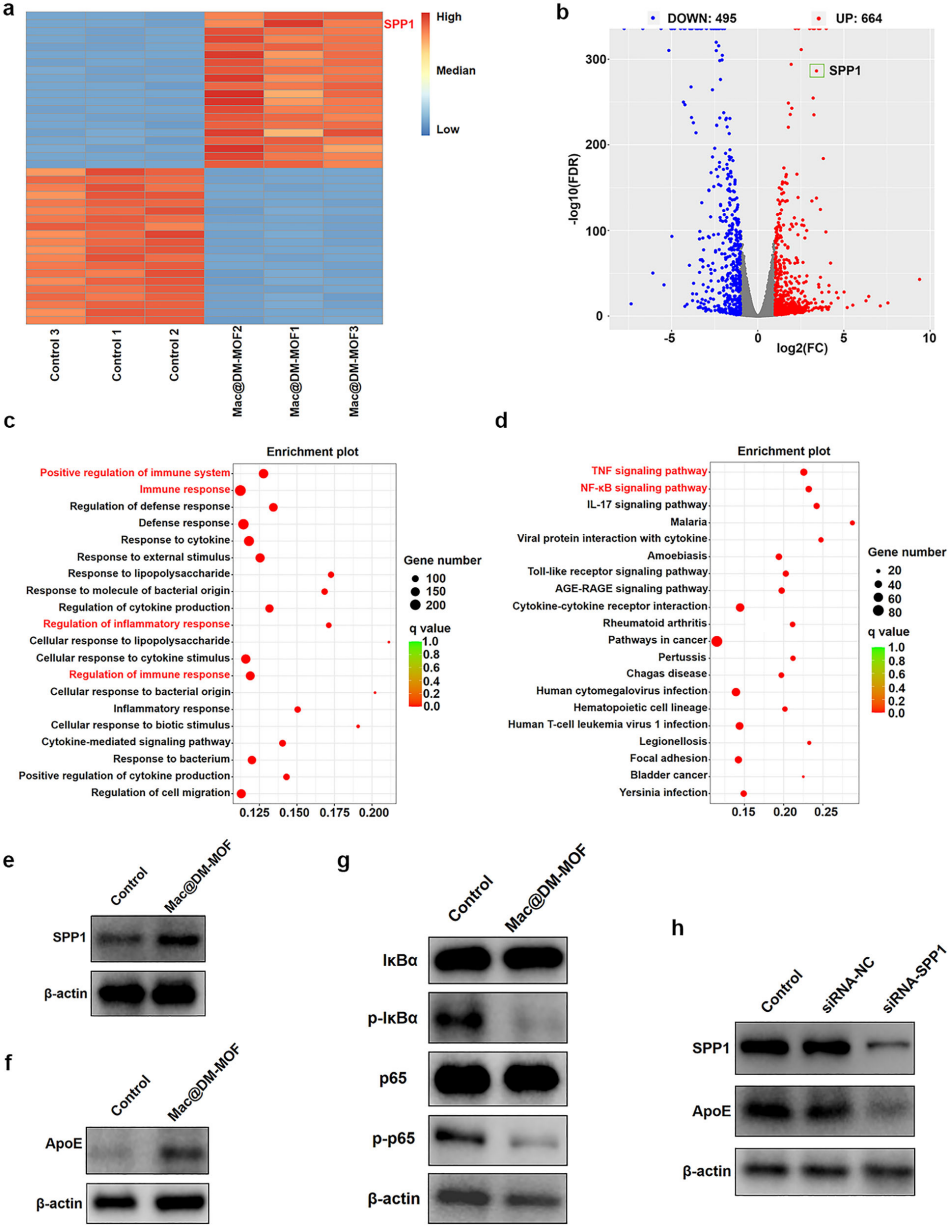
Activation of the SPP1/ApoE signaling pathway is a potential mechanism by which Mac@DM‐MOF exerts its effects. (a). Differential expression gene (DEG) profile of macrophages between the Mac@DM‐MOF treatment and control groups. (b). Volcano plot of DEGs; (c). GO analysis results of DEGs. (d). KEGG pathway analysis results of DEGs. (e). Expression levels of SPP1 in macrophages across different treatment groups. (f). Expression levels of ApoE in macrophages across different treatment groups. (g). NF‐κB signaling pathway activity status in cells across different treatment groups. (h). Impact of SPP1 knockdown on ApoE expression in macrophages.

To elucidate the molecular mechanisms underlying the differences in macrophage polarization between PBS‐treated and Mac@DM‐MOF‐treated groups, macrophages were subjected to high‐throughput RNA sequencing after respective treatments. As illustrated in Figures 5a,b and S13, 664 genes were upregulated, and 495 genes were downregulated in Mac@DM‐MOF‐treated macrophages compared to PBS‐treated cells. Notably, SPP1 was ranked among the top ten significantly upregulated genes. Gene ontology (GO) analysis highlighted alterations in pathways related to the positive regulation of the immune system, immune responses, and inflammatory regulation (Figure 5c). Meanwhile, Kyoto encyclopedia of genes and genomes (KEGG) analysis identified substantial changes in pathways associated with TNF signaling and NF‐κB signaling (Figure 5d). These findings suggest that secreted phosphoprotein 1 (SPP1) may serve as a critical target mediating the immunoregulatory effects of Mac@DM‐MOF.

To investigate further, we assessed the impact of Mac@DM‐MOF on the downstream signaling pathways of SPP1. As shown in Figure 5e,f, the expression levels of SPP1 and its downstream effector Apolipoprotein E (ApoE) were elevated in Mac@DM‐MOF‐treated macrophages. Additionally, the effect of Mac@DM‐MOF on the NF‐κB signaling pathway was examined. Western blot analysis demonstrated a marked inhibition of NF‐κB pathway activity in the Mac@DM‐MOF‐treated group (Figure 5g). To validate the role of SPP1, we constructed an SPP1 small RNA plasmid for macrophage transfection. Targeted knockdown of SPP1 led to an obvious reduction in ApoE levels within the macrophages (Figure 5h). Thus, these findings indicate that the activation of the SPP1/ApoE signaling pathway is likely a key mechanism by which Mac@DM‐MOF exerts its immunoregulatory effects.

### 2.6 Mac@DM‐MOF Induces Macrophage‐Endothelial Communication In Vitro

**FIGURE 6:**
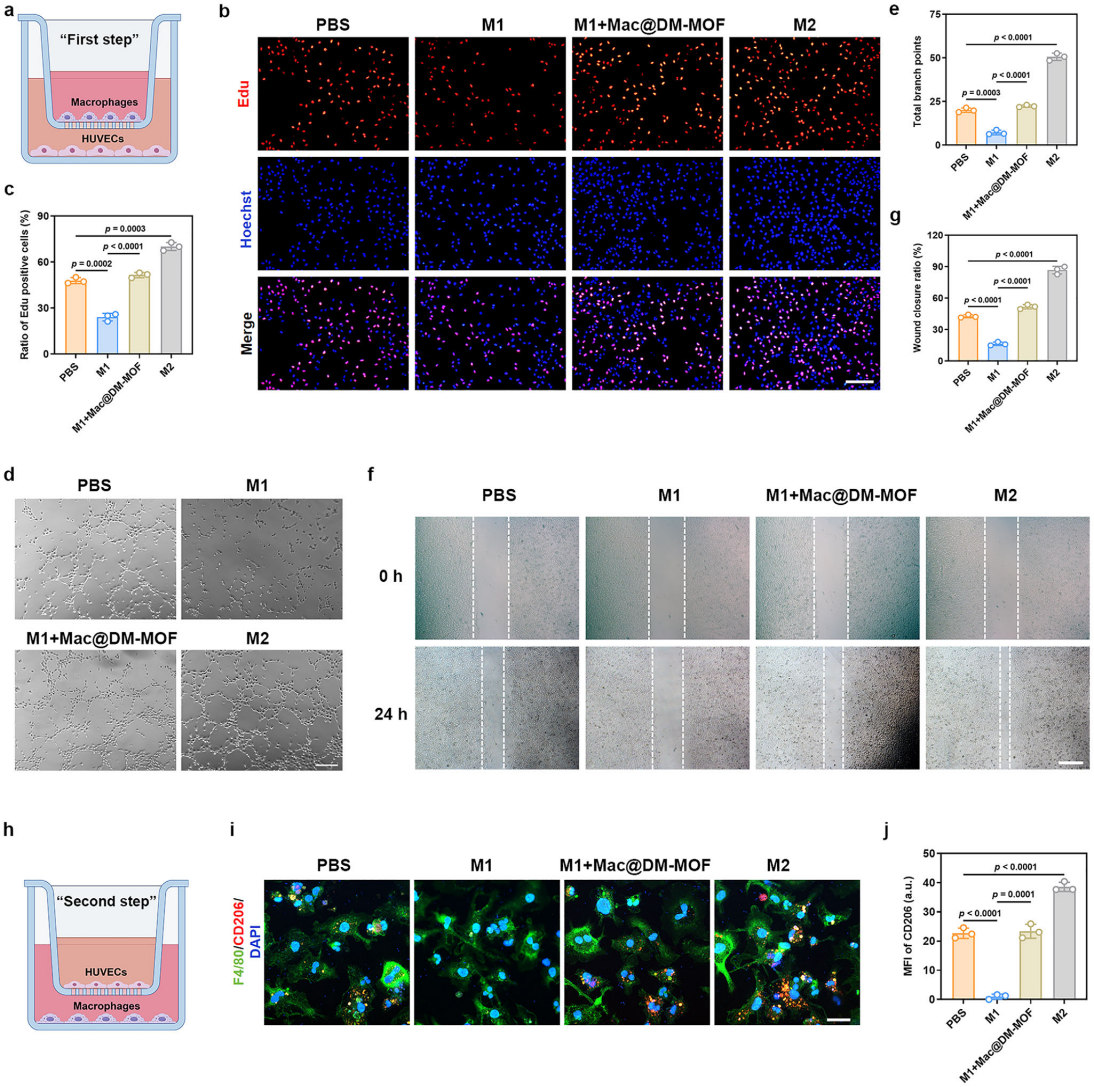
An anti‐inflammatory microenvironment promotes angiogenesis, which in turn reinforces macrophage polarization toward an anti‐inflammatory phenotype. (a). Schematic cell co‐culture model. (b, c). EdU immunofluorescence staining results showing proliferation of HUVECs in the lower chamber under different treatment groups within the co‐culture model, scale bar: 50 µm. (d, e). Evaluation of tube formation ability of cells in the lower chamber across different treatment groups, scale bar: 200 µm. (f, g). Evaluation of migration capacity of cells in the lower chamber across different treatment groups, scale bar: 500 µm. (h). Schematic “second step” cell co‐culture model. (i, j). M2 phenotype polarization of macrophages after re‐co‐culturing with HUVECs collected from different treatment groups in the lower chamber, scale bar: 20 µm.

To further explore the impact of macrophage phenotype shifts and microenvironmental changes on endothelial cell functionality, we employed a “two‐step co‐culture” approach to examine macrophage‐endothelial cell interactions under oxidative stress conditions. In the first step, we established a co‐culture model using a Transwell chamber system, placing macrophages in the upper chamber and HUVECs in the lower chamber. The experimental groups were categorized based on different interventions: Control group, M1 macrophage group, M1 macrophage + Mac@DM‐MOF group, and M2 macrophage group (Figure 6a). After 24 h of co‐culture, HUVECs from the lower chamber were harvested. Results revealed that co‐culture with M1 macrophages impaired HUVEC proliferation (Figure 6b,c), tube formation (Figure 6d,e), and wound closure (Figure 6f,g). Notably, Mac@DM‐MOF treatment effectively mitigated the damage inflicted by M1 macrophages on HUVECs. In the second step of the co‐culture experiment, HUVECs collected from the first step were placed into the upper chamber, while macrophages were seeded into the lower chamber and co‐cultured for an additional 24 h (Figure 6h). Macrophages from the lower chamber were subsequently collected for phenotype marker analysis using immunofluorescence staining (F4/80 and CD206). The results demonstrated that HUVECs pre‐treated with M1 macrophages + Mac@DM‐MOF could restore the anti‐inflammatory capacity of macrophages, as evidenced by an increase in red fluorescence indicating elevated CD206 expression compared to HUVECs treated with M1 macrophages alone (Figure 6i,j).

To further determine whether this bidirectional macrophage‐endothelial communication was mechanistically dependent on the SPP1‐ApoE axis, gain, and loss of function experiments were performed using a five‐group design, including the Control, LPS+IFN‐γ, LPS+IFN‐γ+Mac@DM‐MOF, LPS+IFN‐γ+Mac@DM‐MOF+rOPN and LPS+IFN‐γ+Mac@DM‐MOF+siRNA‐ApoE groups. Recombinant osteopontin (rOPN, 100 ng/mL) was used to activate extracellular SPP1 signaling, whereas siRNA‐ApoE was applied to block the downstream ApoE component of this pathway. Functionally, rOPN markedly enhanced endothelial migration, proliferation, and tube formation in the co‐culture model (Figures S14–S16), indicating that activation of SPP1 signaling promoted the pro‐angiogenic effect of macrophages on endothelial cells. ELISA analysis showed that rOPN significantly decreased the secretion of the pro‐inflammatory cytokines TNF‐α and IL‐6 (Figure S17), while increasing the levels of IL‐10 and TGF‐β1(Figure S18), suggesting a shift toward a reparative immunoregulatory phenotype. At the molecular level, western blot analysis further showed that rOPN increased the expression of SPP1 and ApoE, whereas ApoE knockdown reduced the expression of both proteins. These molecular changes were accompanied by reduced phosphorylation of IκBα and p65, indicating suppression of NF‐κB‐related inflammatory signaling (Figures S19 and S20). Importantly, ApoE knockdown substantially attenuated Mac@DM‐MOF effects, as evidenced by reduced endothelial migration, proliferation, and tube formation, reversal of the cytokine changes, and restoration of IκBα and p65 phosphorylation. These findings indicate that ApoE is functionally required for SPP1‐mediated macrophage‐endothelial communication and further support the conclusion that the SPP1‐ApoE axis is a critical mediator of the immunovascular regulatory effects of Mac@DM‐MOF. Additionally, we simulated a high oxidative stress environment in vitro using H_2_O_2_ and divided the cells into three groups based on treatment conditions: Control group (PBS), H_2_O_2_‐treated group, and H_2_O_2_ + Mac@DM‐MOF‐treated group. EdU immunofluorescence assays revealed that Mac@DM‐MOF alleviated the reduction in HUVEC proliferation caused by oxidative stress (Figure S21). Similarly, Mac@DM‐MOF treatment substantially improved the impaired tube formation and migration capacity of HUVECs under high oxidative stress, ultimately enhancing endothelial cell functionality (Figures S22 and S23). These findings indicate that Mac@DM‐MOF effectively alleviates oxidative stress, reprograms macrophage phenotype polarization, and restores endothelial cell function through intercellular communication. Enhanced endothelial cell function, in turn, establishes a positive feedback loop that further improves macrophage functionality, contributing to the formation of a pro‐healing regulatory circuit between macrophages and endothelial cells. This synergistic interaction emphasizes the therapeutic potential of Mac@DM‐MOF in promoting tissue repair and regeneration under oxidative stress conditions.

### 2.7 Mac@DM‐MOF MNs Promote Wound Healing in Diabetic Mice

**FIGURE 7:**
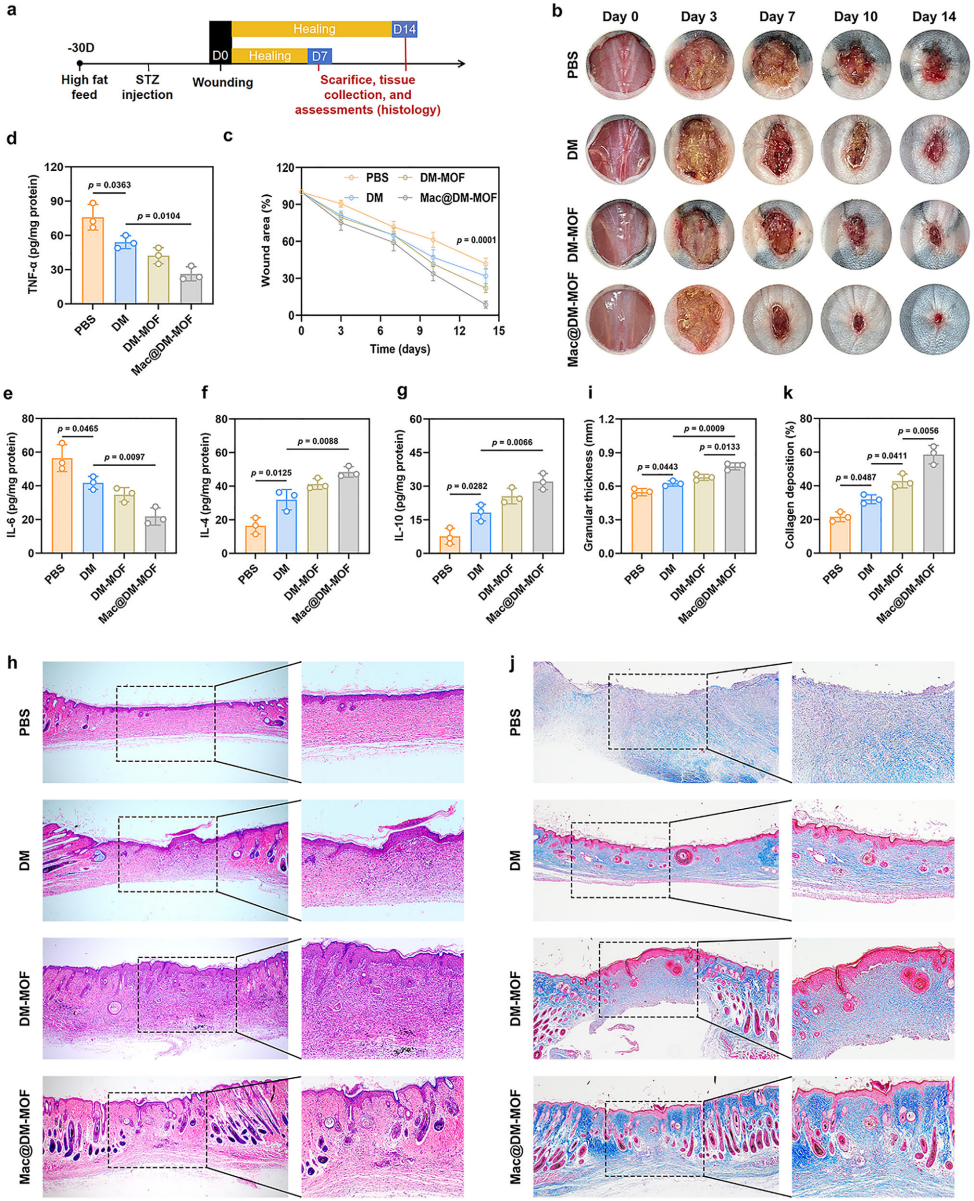
Assessment of the protective effects of Mac@DM‐MOF MNs on wound healing in murine models. (a). Schematic wound model in diabetic mice and the treatment timeline at different time points. (b). Representative images of wound healing in mice across different treatment groups. (c). Wound healing curves for mice in each treatment group. (d, e). Levels of inflammatory cytokines in wound tissues from mice in different treatment groups. (f, g). Levels of anti‐inflammatory cytokines in wound tissues from mice in different treatment groups. (h, i). H&E staining images and corresponding statistical results of wounds on day 14 post‐surgery across treatment groups, scale bar: 400 and 200 µm (insert). (j, k). Masson's trichrome staining images and statistical analysis of wounds on day 14 post‐surgery across treatment groups, scale bar: 400 and 200 µm.

Building upon the promising in vitro results, we assessed the efficacy of the Mac@DM‐MOF MNs in promoting wound healing using a diabetic mouse model. To comprehensively evaluate the therapeutic effects, macroscopic images of wound healing progression were recorded on days 0, 3, 7, 10, and 14 (Figure 7a). These  imgages demonstrated that the Mac@DM‐MOF MN patch accelerated wound closure compared to other treatments (Figure 7b). Quantitative analysis further supported these observations, showing a marked reduction in wound size by day 10, with near‐complete healing achieved by day 14 in the Mac@DM‐MOF MN‐treated group (Figure 7c). Additionally, enzyme‐linked immunosorbent assay (ELISA) analyses of pro‐inflammatory and anti‐inflammatory markers, including TNF‐α, IL‐6, IL‐4, and IL‐10, mirrored the trends observed in vitro (Figure7d‐g). The Mac@DM‐MOF MN patch reduced levels of pro‐inflammatory cytokines (TNF‐α and IL‐6) while enhancing anti‐inflammatory cytokines (IL‐4 and IL‐10), highlighting its capacity to modulate the inflammatory response effectively. This modulation creates a more favorable microenvironment for wound repair by attenuating harmful inflammation and promoting tissue regeneration.

On day 14, histological evaluations, including H&E and Masson's trichrome staining, revealed substantial improvements in tissue regeneration in the Mac@DM‐MOF MN‐treated group. Enhanced granulation tissue formation, characterized by increased thickness and dense collagen deposition, underscored the treatment ability to stimulate robust healing processes (Figure 7h‐k). Importantly, pathological examinations of major organs upon post‐treatment (Figure S24) revealed no significant abnormalities, confirming the good biocompatibility and minimal systemic side effects of the Mac@DM‐MOF MNs. These findings demonstrate the therapeutic potential of the Mac@DM‐MOF MN patch in accelerating wound healing through effective inflammatory modulation and robust tissue regeneration, with minimal adverse effects.

### 2.8 Mac@DM‐MOF MNs Ameliorate Inflammation and Enhance Angiogenesis In Vivo

**FIGURE 8:**
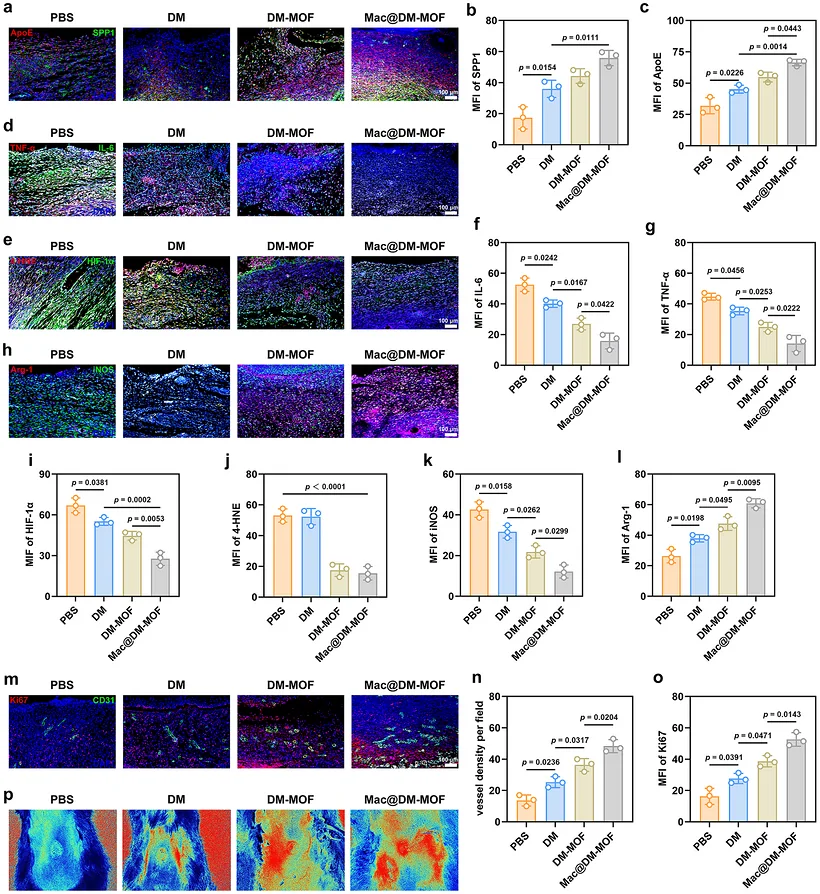
Mac@DM‐MOF MNs create a more conducive healing environment in diabetic wound sites. (a–c). The expression of SPP1 and ApoE in the wound area with indicated treatments. (d–f). Representative images of immunofluorescence staining for evaluating the content of IL‐6 and TNF‐α. (g, i, j). The level of HIF‐1α and 4‐HNE was assessed by immunofluorescent staining. h,k,l. The level of iNOS and Arg‐1 was detected to assess the macrophage phenotype profiles in the wound tissue of each group. (m–o). Representative confocal images of Ki67 and CD31 in tissue specimens from different groups. (p). Representative small animal Doppler imaging of the wound area in each mouse groupon postoperative day 10.

To further explore the inflammatory responses elicited by different treatments, post‐treatment tissue specimens were subjected to detailed analysis. Our results indicated that the intervention with DM obviously increased the expression of SPP1 and ApoE in the diabetic wound tissues, and targeting delivery of DM further enhanced the activity of SPP1/ApoE axis (Figure 8a–c). Improvement of drug administration route effectively inhibited local inflammation responses in the wound area, as seen by the lowest level of IL‐6 and TNF‐α in the Mac@DM‐MOF group (Figure 8d,f,g 8d–f). In terms of the anti‐oxidative property, the results of immunofluorescence staining showed that MOF application significantly ameliorated the ROS burden in the hyperglycemic microenvironment, as witnessed by the level reduction of 4‐HNE which was a biomarker of lipid peroxidation. Moreover, we found that DM treatment alleviated the oxygen deficiency in the wound, which was enhanced by the nano‐system application, as witnessed by the level alteration of HIF‐1α (Figure 8e,i,j 8g,i,j). Notably, the Mac@DM‐MOF MN group exhibited the lowest levels of inducible nitric oxide synthase (iNOS) and the highest levels of SPP1 and inducible nitric oxide synthase (iNOS). These findings suggest the activation of arginase 1 (Arg‐1), which fostered M2 macrophage polarization, providing a more favorable microenvironment for wound healing (Figure 8h,k,l). Immunofluorescence staining of CD31 and Ki67 in wound tissues provided additional insights into the regenerative potential of the treatments. CD31, a marker of endothelial cells, and Ki67, a proliferation marker, revealed varying degrees of angiogenesis and cellular proliferation across treatment groups (Figure 8m–o). Statistical analysis confirmed significant differences, with the Mac@DM‐MOF MN group showing the highest levels of newly vessel formation and Ki67 expression. These results highlight the treatment ability to enhance both vascularization and cellular regeneration‐two essential processes for effective tissue repair.

To further investigate vascular functionality, small animal Doppler imaging was performed on day 10 to assess blood perfusion in the wound area. The results revealed markedly improved blood flow in the Mac@DM‐MOF MN group, correlating with accelerated wound healing compared to other groups (Figures 8p and S18). Enhanced perfusion supports the treatment ability to deliver oxygen and nutrients more effectively, further supporting robust angiogenesis and tissue repair. Collectively, these findings demonstrate that Mac@DM‐MOF MNs not only mitigate harmful inflammation but also promote angiogenesis, cell proliferation, and vascular functionality. This multifaceted therapeutic approach underscores its potential to accelerate wound healing and drive tissue regeneration.

### 2.9 Therapeutic Strategies for Promoting Diabetic Wound Healing in a Porcine Model

**FIGURE 9:**
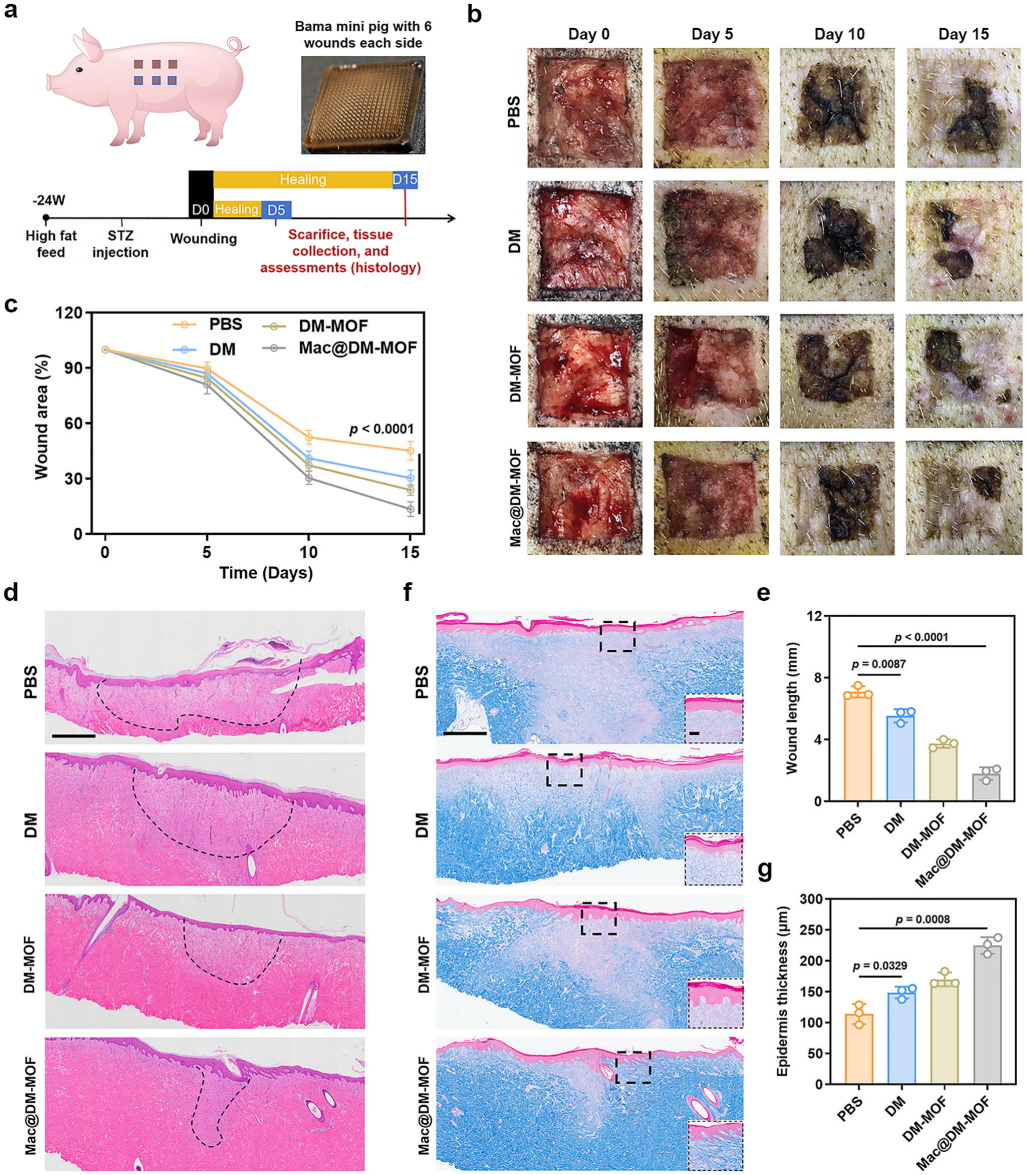
Comprehensive evaluation of wound healing in mini Bama pigs under different treatments. (a). Schematic study design. (b, c). Representative macroscopic images and statistical analysis of wounds on days 0, 5, 10, and 15 across different treatment groups (n=3). (d–e). Representative H&E‐stained images of wounds on day 15, with statistical analysis of wound length for each group, scale bar: 1 mm. (f, g). Representative Masson's trichrome‐stained images of wounds on day 15, with statistical analysis of epidermal thickness for each group, scale bar: 1 mm and 100 µm (inset).

Then, a porcine diabetic wound model was utilized to further evaluate the therapeutic efficacy of Mac@DM‐MOF MNs, due to its close physiological and immunological resemblance to humans, providing a more clinically relevant platform compared to smaller animal models [30, 31]. This model enabled detailed analysis of therapeutic outcomes and mechanisms, yielding robust data applicable to human clinical settings. The wound healing process was monitored at critical time points: days 0, 5, 10, and 15 (Figure 9a). Macroscopic evaluation of the wounds (Figure 9b) revealed distinct healing dynamics across treatment groups. Quantitative analysis demonstrated significant differences in healing rates, with the Mac@DM‐MOF MN group showing notably smaller wound areas by day 10 compared to other treatments. By day 15, the Mac@DM‐MOF MN‐treated wounds exhibited near‐complete closure, underscoring the superior efficacy of this intervention (Figure 9c).

To further assess the quality of tissue repair, histological examinations were performed on day 15. H&E staining and Masson's trichrome staining provided detailed insights into wound morphology, epidermal regeneration, and dermal remodeling, focusing on wound length, epidermal thickness, and the development of rete ridges‐key indicators of effective healing (Figure 9d,f). Statistical analyses confirmed that Mac@DM‐MOF MN treatment resulted in much improved outcomes in all parameters, with enhanced tissue architecture and robust healing compared to other groups (Figures 9e,g and S26). These findings highlight the critical role of Mac@DM‐MOF MNs in promoting efficient wound closure, improving tissue morphology, and supporting overall regeneration in a diabetic porcine model. The results underscore the potential of this therapeutic approach for advancing wound care in clinical applications.

## 3 Conclusion

This study presents Mac@DM‐MOF, an innovative nanozyme platform designed as a multifunctional therapeutic strategy to address the dual challenges of oxidative stress and immune dysregulation in diabetic wound environments. Through comprehensive functional design and characterization, we demonstrate its enhanced catalytic activity, structural stability, and seamless integration of macrophage membranes, establishing a robust foundation for wound microenvironment modulation. In diabetic mouse and Bama mini pig models, the macrophage membrane‐coated nanosystem effectively scavenges ROS, alleviates inflammation, and restores vascular function. Notably, this vascular restoration further reinforces M2 macrophage polarization, creating a self‐sustaining pro‐healing feedback loop. These findings highlight macrophage‐endothelial crosstalk as a critical regulatory mechanism in wound repair, and propose a promising strategy to simultaneously resolve chronic inflammation and promote angiogenesis in diabetic wounds.

The macrophage membrane coating of DM‐MOF (Mac@DM‐MOF) uniquely positions this platform as an immunomodulatory agent. Macrophage polarization assays reveal the ability of Mac@DM‐MOF to effectively reprogram macrophages toward an M2 phenotype, counteracting the pro‐inflammatory M1 state [32, 33]. Flow cytometry and immunofluorescence staining consistently demonstrate that Mac@DM‐MOF outperformed its counterparts (DM and DM‐MOF) in enhancing M2 marker expression while suppressing M1‐associated phenotypes. These results align with the overarching therapeutic goal of restoring immune balance in chronic diabetic wounds. The incorporation of DM further enhances the bioactivity of the system, enabling a synergistic effect that combines ROS scavenging with immune modulation to mitigate chronic inflammation‐a central feature of diabetic wound pathology [34].

Mechanistic investigations illuminate the crucial role of the SPP1/ApoE signaling axis in mediating the therapeutic effects of Mac@DM‐MOF, highlighting its intricate molecular mechanisms. Comprehensive RNA sequencing coupled with pathway enrichment analyses unveil a robust upregulation of SPP1 and its associated immune‐regulatory networks, particularly involving TNF and NF‐κB signaling pathways. Notably, the activation of SPP1 is shown to enhance ApoE expression, thereby initiating a positive feedback loop that mitigates pro‐inflammatory signaling and facilitates tissue repair and regeneration. This SPP1‐mediated modulation of ApoE expression is central to anti‐inflammatory efficacy of the nanosystem, emphasizing its potential to attenuate the immune response in pathological environments. Furthermore, the inhibition of NF‐κB activation reinforces the anti‐inflammatory action of Mac@DM‐MOF, highlighting its capacity to modulate key transcription factors involved in immune response and inflammation resolution. Given that inflammation plays a central role in the pathogenesis of numerous diseases, including autoimmune disorders, cardiovascular diseases, and neurodegenerative conditions [35, 36], this mechanism opens new avenues for the development of targeted therapeutic strategies aimed at rebalancing immune responses. The findings from this study not only reinforce the sophistication of Mac@DM‐MOF as a promising therapeutic platform, but also highlight the impact of the SPP1/ApoE signaling axis as a central regulatory node in the modulation of chronic inflammation and tissue regeneration.

The multifunctionality of Mac@DM‐MOF extends beyond oxidative stress reduction and immune modulation to include restoration of vascular function. By targeting the interplay between macrophages and endothelial cells, Mac@DM‐MOF promotes angiogenesis‐a critical process for effective wound healing. The macrophage membrane coating enhances the therapeutic delivery of DM‐MOF while minimizing potential immunogenicity, reflecting a biomimetic strategy that ensures biocompatibility and targeted action. While this study presents promising findings, certain limitations warrant further investigation to fully realize the potential of Mac@DM‐MOF. A limitation of the present study is that the stability of Mac@DM‐MOF was not directly assessed in protease‐rich or ROS‐enriched wound‐mimicking conditions. Although the revised physicochemical characterization supports successful membrane coating and basic colloidal stability, further studies are warranted to determine whether the membrane layer remains intact in the hostile diabetic wound microenvironment before reaching target cells. Although the SPP1/ApoE signaling axis is identified as a key mechanism driving its therapeutic effects, other signaling pathways may contribute to its multifaceted activity and deserve comprehensive exploration. Delineating these pathways could provide a more complete understanding on the molecular underpinnings of the therapeutic efficacy. In addition, the macrophage membranes used for coating were prepared from RAW264.7 cells without further characterization of polarization‐associated markers. Therefore, the present study cannot exclude the possibility that the activation state of the source cells may have influenced the biological behavior of the membrane‐coated system. Future studies are warranted to compare membranes derived from different macrophage phenotypes, particularly M2‐polarized macrophages, to further optimize the immunomodulatory performance of this platform. Future research should focus on rigorous clinical validation in diabetic wound models, emphasizing critical endpoints such as wound closure, re‐epithelialization, and neovascularization. These studies will be pivotal in bridging the gap between preclinical success and clinical application, ensuring that Mac@DM‐MOF is optimized for therapeutic use. Additionally, longitudinal in vivo investigations are essential to evaluate the long‐term safety, biocompatibility, and sustained therapeutic efficacy, particularly under chronic use scenarios. Beyond diabetic wounds, the potential of Mac@DM‐MOF to address other chronic inflammatory conditions, such as rheumatoid arthritis and atherosclerosis, represents a compelling avenue for future research [37, 38]. Its ability to modulate oxidative stress, immune responses, and vascular function suggests that it could serve as a versatile therapeutic strategy across diverse pathological contexts [39]. Expanding its application in these domains could enhance its clinical utility and broader impact on inflammatory disease management.

Thus, Mac@DM‐MOF MNs represent a transformative advancement in nanozyme therapeutics, introducing macrophage‐endothelial crosstalk as a therapeutic target for diabetic wound healing. By integrating structural innovation with biomimetic design, this platform effectively addresses the dual pathological hallmarks of oxidative stress and immune dysregulation. The activation of the SPP1/ApoE axis underscores the mechanistic sophistication and therapeutic potential of Mac@DM‐MOF MNs, positioning it as a versatile intervention for chronic wound management. While further studies are necessary to facilitate clinical translation, this work establishes a robust foundation for next‐generation nanozyme development, with broad implications for the treatment of chronic inflammatory diseases.

## Conflicts of Interest

The authors declare no conflicts of interest.

## Supporting information




**Supporting file**: adhm71456‐sup‐0001‐SuppMat.docx.

## Data Availability

The data that support the findings of this study are available from the corresponding author upon reasonable request.
