# Supplementary material for: Macrophage Membrane‐Camouflaged Nanozyme Microneedles Restore Immunovascular Homeostasis Through SPP1‐ApoE Signaling in Diabetic Wounds
Source: Adv Healthc Mater. 2026 Jul 26;15(31):e71456. doi: 10.1002/adhm.71456 (PMC13495865; doi:10.1002/adhm.71456)
Supplement: Supplementary file 1 [file ADHM-15-0-s001.docx]

Supplementary Information

Macrophage Membrane-Camouflaged Nanozyme Microneedles Restore Immunovascular Homeostasis through SPP1-ApoE Signaling in Diabetic Wounds

Wu et al.

Supplementary Experimental Section

**Methods:** Transmission electron microscopy (TEM) was conducted using a JEOL JEM 2100Plus microscope (Japan) and an FEI 300 kV Titan spherical aberration-corrected electron microscope (USA). Powder X-ray diffraction (XRD) analysis was performed on a Bruker D8 Discover XRD system (Germany). Ultraviolet-visible (UV-vis) absorption spectra were acquired with a Shimadzu UV-3600 spectrophotometer (Japan), while fluorescence spectra were measured on a Horiba Fluoromax spectrophotometer (Japan). Measurements of hydrodynamic sizes and zeta potentials were conducted with a Malvern Zetasizer Nano ZS (UK). Fourier-transform infrared (FT-IR) spectra were obtained using a Shimadzu IR Prestige-21 FTIR spectrometer (Japan). X-ray photoelectron spectroscopy (XPS) data were recorded using a SPECSLab system (Germany).

**Preparation of macrophage membrane**：Macrophage membranes were isolated from RAW264.7 cells using a membrane protein extraction kit (Beyotime, China) following the manufacturer’s instructions. Briefly, cells were incubated in pre-cooled membrane protein extraction buffer for 15 min and then gently disrupted by 10–20 strokes in a Dounce homogenizer. The homogenate was first centrifuged at 800 ×g for 10 min to remove nuclei and residual intact cells, after which the supernatant was further centrifuged at 15,000 ×g for 30 min to harvest the membrane fraction. The protein concentration of the collected membrane pellet was determined using a BCA protein assay. Subsequently, the membrane fraction was sonicated in an ultrasonic bath for 15 min and extruded 11 times through a 400 nm polycarbonate porous membrane using an Avanti mini-extruder (Avanti, Canada) to obtain macrophage membrane vesicles.

**Synthesis of DM-MOF and DM-MOF-HA:** The synthesis of DM-MOF nanoparticles was carried out with the support of Changsha Kemeida BioTech Co., Ltd. Briefly, zinc nitrate hexahydrate (Zn(NO₃)₂·6H₂O) and copper nitrate trihydrate (Cu(NO₃)₂·3H₂O) were dissolved in methanol, while 2-methylimidazole was separately dissolved in methanol. The two solutions were subsequently mixed and allowed to react at room temperature for 3 hours. After the reaction, the resulting product was collected via centrifugation, washed three times with dimethylformamide (DMF), and then dispersed in DMF for further use. To prepare DM-MOF, the MOF was mixed with the drug (mass ratio 1:1) and stirred at room temperature overnight. The mixture was then centrifuged, washed three times with DMF, followed by three washes with water, yielding DM-MOF. Subsequently, DM-MOF was combined with hyaluronic acid (HA) in water and stirred at room temperature overnight. After centrifugation and three additional washes with water, DM-MOF-HA was obtained.

**Synthesis of Mac@DM-MOF**: Mac@DM-MOF was synthesized with a sonication method. Briefly, the macrophage membrane was mixed with the nanoparticles at a membrane-to-polymer weight ratio of 1:1. Then, the mixture was sonicated with a bath sonicator for 2 min to complete the membrane coating. The excess membrane was removed by centrifuging for 10 min and rinsing with PBS for 3 times.

**Synthesis of Mac@DM-MOF MNs:** The polydimethylsiloxane mold for the microneedle patch (MN) features a 20 × 20 array of microneedles, each with a height of 600 μm and a substrate diameter of 250 μm (Engineering for Life, China). Mac@DM-MOF was mixed with a 30% (w/v) HA solution and stirred for 20 min to obtain the tip solution. This HA-Mac@DM-MOF mixture was then placed into the MN mold, with vacuum pumping used to fill the cavities of the template. Any air bubbles on the surface and excess HA solution were removed, and a 25% (w/v) polyvinyl alcohol (PVA) solution was added to form the base of the MN patch. The template was dried at 37°C for 36 h, then demolded and stored at 4°C for subsequent use. The morphology of the Mac@DM-MOF MNs was examined using a stereomicroscope and scanning electron microscope. For skin penetration assessment, the murine dorsal skin was subjected to pressure from the Mac@DM-MOF MNs, followed by tissue collection for trypan blue and hematoxylin and eosin (H&E) staining. To evaluate mechanical strength, the MNs were placed on a rigid stainless-steel platform, and a sensor was moved toward the MNs at a rate of 0.1 mm/s. Changes in needle force were recorded during this process.

**Sample collection:** All animal procedures were approved by the Institutional Animal Care and Use Committees at Tongji Medical College, Huazhong University of Science and Technology (IACUC Number: 4003) and Hubei Yizhicheng Biotechnology Co., Ltd (IACUC Number: 202402008).

For the mouse study, male C57BL/6J mice (6-8 weeks, 18-20 g) were housed under specific pathogen-free conditions with a 12-hour light/dark cycle. Mice were divided into two groups: high-fat diet (HFD) and normal chow diet. After 4 weeks, HFD mice were intraperitoneally injected with streptozotocin (STZ, 60 mg/kg/day) for 3 consecutive days. Blood glucose levels were monitored using a glucose meter, and diabetes was confirmed when levels exceeded 16.7 mM. Mice were then anesthetized, and full-thickness skin defects (10 mm diameter) were created. Wound tissues were collected on postoperative day 14, fixed in 4% paraformaldehyde, and processed for immunofluorescence staining.

In the Bama mini pig model, male pigs (30-40 kg, 1-2 years old) were purchased from Hubei Yizhicheng Biotechnology Co., Ltd. Diabetes was induced by intravenous STZ injection (75 mg/kg). Pigs with fasting glucose ≥11.1 mmol/L sustained for >3 days after the second injection were considered diabetic. Anesthesia was induced with midazolam and xylazine hydrochloride (0.14 mL/kg) and maintained with inhaled isoflurane during surgery. Dermal full-thickness wounds (approximately 2 cm × 2 cm) were created using a scalpel, and wounds were treated with PBS, DM, DM-MOF, or Mac@DM-MOF. After treatment, wounds were covered with 3M Tegaderm Transparent Film. Glucose levels were monitored regularly, and wound images were captured on days 5, 10, and 15 post-surgery. Wound areas were measured as previously described^[1]^. On day 15, wound tissues were harvested, fixed in 4% paraformaldehyde, and processed for immunofluorescence staining.

**Transcriptome sequencing:** Total RNA was isolated from cells using Trizol Reagent (Thermo Fisher Scientific, USA), and its concentration, quality, and integrity were evaluated with a NanoDrop spectrophotometer (Thermo Fisher Scientific, USA). RNA integrity was further assessed using the Qsep400 instrument. RNA libraries were constructed using the VAHTS mRNA-seq V8 Library Prep Kit for Illumina, with total RNA (1 μg) as input. The library preparation process involved polyA selection, RNA fragmentation, reverse transcription using random hexamers, and 150-nt paired-end sequencing on the Illumina Novaseq 6000 platform. Low-quality reads and adapters were removed using cutadapt (version 1.11), and clean reads were mapped to the reference genomes using Hisat2 (version 2.1.0), with a maximum of two mismatches. Gene annotations were obtained by aligning the identified genes to the NR (RefSeq non-redundant) protein database. Transcript abundance was quantified and normalized as FPKM (Fragments Per Kilobase of transcript per Million Mapped Reads) using FeatureCount (version 1.6.0)^[2]^. Differential gene expression was assessed with edgeR, applying a threshold of FDR < 0.05 and |log2FoldChange| > 1. GO (Gene Ontology)^[3]^ and KEGG (Kyoto Encyclopedia of Genes and Genomes)^[4]^ enrichment analyses were performed using the ClusterProfiler R package (http://www.bioconductor.org/packages/release/bioc/html/clusterProfiler.html)^[5]^, with enrichment significance determined by hypergeometric distribution and a q-value cutoff of 0.05.

**Coomassie brilliant blue staining:** Membrane proteins were extracted from macrophages using a membrane and cytosol protein extraction kit (Beyotime, China). Protein samples (40 μg each) were loaded onto a 10% SDS-PAGE gel, and electrophoresis was performed for 1 h. After separation, the gel was washed three times with PBS. The proteins were then stained with Coomassie Brilliant Blue solution (20 mL, Servicebio, China) at room temperature for 30 min. Following staining, the solution was removed, and the gel was washed three times with PBS for 10 min. The stained gel image was captured using a stereomicroscope (Olympus, Japan).

**Cell culture:** Macrophages were isolated from the bone marrow of male C57BL/6J mice (6 weeks old). Bone marrow was flushed from the femurs and layered onto Percoll reagent, followed by density gradient centrifugation according to the manufacturer’s instructions. After three washes, macrophages were cultured in RPMI 1640 medium supplemented with 10% FBS and penicillin/streptomycin (100 U/mL). Human umbilical vein endothelial cells (HUVECs) were obtained from the Cell Bank of the Chinese Academy of Sciences (China) and cultured in RPMI 1640 medium with 10% FBS and penicillin/streptomycin (100 U/mL).

**Macrophage phenotype examination:** Macrophages pre-treated with IL-4+IL-13 or LPS+IFN-γ were allocated into four groups: PBS, DM, DM-MOF, and Mac@DM-MOF. The expression levels of phenotype markers F4/80, CD206, and CD86 were analyzed through immunofluorescence staining and flow cytometry. For immunofluorescence, cells were first collected, washed in PBS, and fixed with 4% paraformaldehyde for 10 minutes. After washing three times with PBS, cells were permeabilized on ice with 0.25% Triton X-100 for 10 minutes and subsequently blocked with goat serum for 30 minutes. Primary antibodies targeting F4/80 (Abclonal, 1:500, USA) and CD206/CD86 (Abclonal, 1:500, USA) were added, and the cells were incubated at 4 °C overnight. Following three washes with PBST, cells were stained with FITC- and Cy3-conjugated secondary antibodies at room temperature for 1 hour, shielded from light. After further washes and DAPI staining, fluorescence imaging was performed using confocal laser scanning microscopy.

To evaluate the influence of HUVECs on macrophage polarization, macrophages were co-cultured with HUVECs and grouped according to the treatments administered to HUVECs: PBS, M1 macrophages, M1 macrophages+Mac@DM-MOF, and M2 macrophages. After a 12-hour treatment period, cells were collected and marker expression was assessed. For flow cytometry, following immunofluorescence staining, macrophages were collected, washed, and resuspended in blocking buffer with 2% BSA to minimize nonspecific binding. Cells were incubated with CD11b-FITC, F4/80-APC, CD86-PE, and CD206-PerCP (BioLegend, USA) at 4 °C for 30 minutes in the dark. After incubation, cells were washed twice with PBS, fixed in 1% paraformaldehyde, and analyzed on a BD FACSCanto II flow cytometer (BD Biosciences, USA) with FlowJo software for data analysis.

**EdU cell proliferation assays:** Cell proliferation was assessed using the EdU assay kit (Beyotime, China). HUVECs were plated in 24-well plates at a density of 2 X10^5^ cells per well and divided into four or three treatment groups, respectively: PBS, M1 macrophages, M1 macrophages+Mac@DM-MOF, and M2 macrophages; or PBS, H_2_O_2_, and H_2_O_2_+Mac@DM-MOF. After 24 hours of treatment, HUVECs in the lower chamber were stained with EdU reagent, following the manufacturer’s protocol, to evaluate cell proliferation.

**Scratch wound assay:** HUVECs were cultured in the lower chamber of a 6-well plate. Once cells reached approximately 90% confluence, a scratch was created using a sterile 1 mL micropipette tip. Following PBS washes to remove debris, serum-free medium was added to the lower chamber. The upper chamber, equipped with 0.45 μm pore filters, was treated with one of several reagents: PBS, M1 macrophages, M1 macrophages+Mac@DM-MOF, and M2 macrophages; or PBS, H_2_O_2_, and H_2_O_2_+Mac@DM-MOF. Images of the scratch were captured at 0 h and 24 h, and the scratch closure percentage was quantified using ImageJ software.

**Tube formation assay:** Each well of a 96-well culture plate was coated with Matrigel (50 μL, Corning, USA) and evenly spread on ice. The plate was then incubated at 37 °C for 30 minutes to allow the Matrigel to solidify. HUVECs were seeded in a 6-well plate at a density of 4 × 10^5^ cells per well and allocated to four or three treatment groups, respectively: PBS, M1 macrophages, M1 macrophages+Mac@DM-MOF, and M2 macrophages; or PBS, H_2_O_2_, and H_2_O_2_+Mac@DM-MOF. After 24 hours of treatment, the cells were collected and seeded onto the Matrigel-coated 96-well plate. Capillary-like tube formation was observed under an optical microscope 6 hours post-seeding.

**ROS detection:** Intracellular ROS levels in macrophages were measured using the DCFH-DA probe (Beyotime, China) following the manufacturer’s protocol. Macrophages were divided into the following treatment groups: PBS, H_2_O_2_, H_2_O_2_+DM, H_2_O_2_+DM-MOF, and H_2_O_2_+Mac@DM-MOF. After 12 hours of treatment, cells were harvested and rinsed twice with PBS. Macrophages were then incubated with DCFH-DA (10 μM) at 37 °C for 20 minutes in the dark. Post-incubation, cells were washed three times with serum-free medium, and mean fluorescence intensity was quantified using ImageJ software.

**CCK-8 assay:** Macrophages or HUVECs were seeded at a density of 5 × 10^3^ cells per well in 96-well culture plates and divided into four treatment groups: PBS, DM, DM-MOF, and Mac@DM-MOF. After 24 hours of treatment, CCK-8 reagent (10 μL, Boster, China) was added to each well. Absorbance was measured at 450 nm using a spectrophotometer (BioTek, USA) to evaluate cell viability.

**Live/dead staining:** Macrophages or HUVECs were seeded in 96-well plates and exposed to treatments with PBS, DM, DM-MOF, or Mac@DM-MOF at 37 °C for 24 hours. Following treatment, cells were stained with calcein-acetoxymethyl ester (Calcein-AM) and propidium iodide (PI) (Beyotime, China) in the dark for 30 min. Live and dead cells were then observed under a fluorescence microscope.

**Mouse skin wound model:** Male C57BL/6J mice (6–8 weeks old) were maintained under specific pathogen-free conditions and subjected to a high-fat diet combined with intraperitoneal STZ injection to establish a diabetic model. Once diabetes was confirmed, a full-thickness excisional skin wound (1.0 × 1.0 cm) was created on each mouse. Mice were then randomly assigned to one of four treatment groups: PBS, DM, DM-MOF MNs, and Mac@DM-MOF MNs. Treatments were applied on days 0, 3, 5, 7, 9, and 11 post-surgery. Wound healing was monitored by digital imaging on days 0, 3, 7, 10, and 14 post-wounding. After 14 days, mice were euthanized, and wound tissues along with organs were collected for further analysis.

**Blood flow assessment at wound site:** On post-operative day 10, blood flow in the wound area was evaluated using a laser speckle contrast imaging system (PERIMED Ltd, Stockholm, Sweden). The mean perfusion units (MPU) ratio was calculated by comparing the MPU of the wound region (ROI-1) to that of the adjacent area (ROI-2).

**ELISA assay:** Levels of TNF-α, IL-6, IL-4, and IL-10 in mouse wound tissues were quantified using ELISA kits (Boster, China), following the manufacturer’s instructions.

**Western blot:** Membrane proteins were extracted from macrophages using a membrane and cytosol protein extraction kit (Beyotime, China), while total proteins were isolated with RIPA lysis buffer (Beyotime, China). Lysates were centrifuged at 12,000 g at 4 °C for 10 minutes, and protein concentrations were determined using a BCA assay. Equal amounts of protein (30 μg per sample) were separated on 10% SDS-PAGE and transferred to a PVDF membrane. Membranes were incubated overnight at 4 °C with primary antibodies, including SPP1, ApoE, IκBα, p-IκBα, p65, and p-p65 (Abclonal, 1:1000, China). Following three washes with TBST, membranes were incubated with horseradish peroxidase-conjugated secondary antibodies at room temperature for 1 hour. Protein bands were visualized using a LiDE110 scanner (Canon, Japan).

**Statistical analysis:** Data are presented as mean ± SD. Statistical significance was determined using a two-tailed Student’s t-test, performed in Excel.

**Fig. S1.The force-displacement curve of Mac@DM-MOF MNs**.

**Fig. S2. The release curve of DM from the drug delivery nanoparticles within indicated condition.**

**Fig. S3. Cytotoxicity assay detected by CCK-8.** CCK-8 assay of HUVECs (a) and BMDMs (b) after different treatments: PBS, DM, DM-MOF and Mac@DM-MOF.

**Fig. S4.** Cytotoxicity assay of HUVECs analyzed via immunofluorescence. Representative live/dead staining images of HUVECs following various treatments: PBS, DM-MOF, and Mac@DM-MOF. Dead cells are shown in red, and live cells in green. Scale bar: 200 μm.

**Fig. S5.** Cytotoxicity assay of BMDMs analyzed via immunofluorescence. Representative live/dead staining images of HUVECs following various treatments: PBS, DM-MOF, and Mac@DM-MOF. Dead cells are shown in red, and live cells in green. Scale bar: 200 μm.

**Fig. S6.** Comparison of F4/80(+) and CD206(+) cells under different treatments. BMDMs were treated with IL-4, IL-13, and various nanomaterials, followed by assessment of phenotype markers using flow cytometry.

**Fig. S7.** Comparison of F4/80(+) and CD86(+) cells under different treatments. BMDMs were treated with LPS, IFN-γ, and various nanomaterials, followed by assessment of phenotype markers using flow cytometry.

**Fig. S8.** Comparison of F4/80(+) and CD206(+) cells under different treatments. BMDMs were treated with LPS, IFN-γ, and various nanomaterials, followed by assessment of phenotype markers using flow cytometry.

**Fig. S9.** Comparison of the fluorescence intensity of CD206 in BMDMs under different treatments. BMDMs were treated with IL-4, IL-13, and various nanomaterials, followed by assessment of phenotype markers using immunofluorescence.

**Fig. S10.** Comparison of the fluorescence intensity of CD86 in BMDMs under different treatments. BMDMs were treated with LPS, IFN-γ, and various nanomaterials, followed by assessment of phenotype markers using immunofluorescence.

**Fig. S11.** Comparison of the fluorescence intensity of CD206 in BMDMs under different treatments. BMDMs were treated with LPS, IFN-γ, and various nanomaterials, followed by assessment of phenotype markers using immunofluorescence.

**Fig. S12. Comparison of the inflammatory factors in BMDMs under different treatments.** BMDMs were treated with H_2_O_2_, DM, and other nanomaterials, followed by assessment of IL-6 (a) and TNF-α (b) using ELISA assays.

**Fig. S13.** Protein-protein interaction network from high-throughput sequencing of macrophages in different treatment groups. Macrophages were treated with PBS or Mac@DM-MOF, collected for high-throughput sequencing, and subsequently analyzed for protein interactions.

**Fig.S14**. **Modulation of the SPP1-ApoE axis regulates HUVEC migration in the macrophage-endothelial co-culture model.** Representative images of migrated HUVECs in different treatment groups. Scale bar: 50 μm.

**Fig.S15.** **Modulation of the SPP1-ApoE axis regulates HUVEC proliferation in the macrophage-endothelial co-culture model.** HUVEC proliferation was assessed by EdU staining in different treatment groups. Scale bar: 100 μm.

**Fig.S16. Modulation of the SPP1-ApoE axis regulates HUVEC tube formation in the macrophage-endothelial co-culture model.** Representative images of HUVEC tube formation in different treatment groups. Scale bar: 100 μm.

**Fig.S17. Modulation of the SPP1-ApoE axis regulates inflammatory cytokine secretion in the macrophage-endothelial co-culture model.** Culture supernatants from different treatment groups were collected, and the levels of TNF-α (a) and IL-6 (b) were determined by ELISA with corresponding statistical analysis.

**Fig.S18. Modulation of the SPP1-ApoE axis regulates anti-inflammatory cytokine secretion in the macrophage-endothelial co-culture model.** Culture supernatants from different treatment groups were collected, and the levels of IL-10 (a) and TGF-β1 (b) were determined by ELISA with corresponding statistical analysis.

Fig.S19. **Impact of rOPN stimulation and ApoE knockdown on the SPP1-ApoE axis in macrophages from the macrophage-endothelial co-culture model.** Expression levels of SPP1 and ApoE in macrophages across different treatment groups.

**Fig.S20. Modulation of the SPP1-ApoE axis regulates NF-κB-related signaling in macrophages from the macrophage-endothelial co-culture model.** NF-κB signaling pathway activity status in macrophage across different treatment groups.

**Fig. S21. Mac@DM-MOF restores BMDM proliferation under oxidative stress.** BMDMs were treated with PBS, H_2_O_2_, or H_2_O_2_ + Mac@DM-MOF, and proliferation was assessed using EdU assays (a) with corresponding statistical analysis (b). Scale bar: 50 μm.

**Fig. S22. Mac@DM-MOF restores tube formation of HUVECs under oxidative stress.** HUVECs were treated with PBS, H_2_O_2_, or H_2_O_2_ + Mac@DM-MOF, and proliferation was assessed using tube formation assays (a) with corresponding statistical analysis (b). Scale bar: 200 μm.

**Fig. S23.** Mac@DM-MOF restores migration ability of HUVECs under oxidative stress. HUVECs were treated with PBS, H_2_O_2_, or H_2_O_2_ + Mac@DM-MOF, and proliferation was assessed using cell scratch assays. Scale bar: 500 μm.

**Fig. S24.** Biosafety assessment following MN application. H&E staining images of major organs (lung, liver, spleen, kidney, and heart) from mice with diabetic wound models treated with different MNs for 14 days.

**Fig. S25.** Blood perfusion analysis following MN application in diabetic mice. Statistical results from small animal Doppler imaging of blood perfusion in diabetic wound models treated with different MNs for 10 days.

**Fig. S26.** Statistical analysis of Masson’s trichrome staining in wound tissues from diabetic Bama mini pigs on day 14 post-surgery across treatment groups. Wound tissues from different treatment groups were collected 14 days after surgery, stained, and analyzed for Rete ridge statistics.

**Supplementary References**

[1]	T. Wei, T. Pan, X. Peng, M. Zhang, R. Guo, Y. Guo, X. Mei, Y. Zhang, J. Qi, F. Dong, M. Han, F. Kong, L. Zou, D. Li, D. Zhi, W. Wu, D. Kong, S. Zhang, C. Zhang, Janus liposozyme for the modulation of redox and immune homeostasis in infected diabetic wounds. *Nat. Nanotechnol.* **2024**, *19*, 1178-1189.

[2]	G. Yu, L. G. Wang, Y. Han, Q. Y. He, clusterProfiler: an R package for comparing biological themes among gene clusters. *Omics* **2012**, *16*, 284-287.

[3]	M. Ashburner, C. A. Ball, J. A. Blake, D. Botstein, H. Butler, J. M. Cherry, A. P. Davis, K. Dolinski, S. S. Dwight, J. T. Eppig, M. A. Harris, D. P. Hill, L. Issel-Tarver, A. Kasarskis, S. Lewis, J. C. Matese, J. E. Richardson, M. Ringwald, G. M. Rubin, G. Sherlock, Gene ontology: tool for the unification of biology. *Nat. Genet.* **2000**, *25*, 25-29.

[4]	M. Kanehisa, S. Goto, KEGG: kyoto encyclopedia of genes and genomes. *Nucleic Acids Res.* **2000**, *28*, 27-30.

[5]	B. Li, C. N. Dewey, RSEM: accurate transcript quantification from RNA-Seq data with or without a reference genome. *BMC Bioinformatics* **2011**, *12*, 323.
